# Automatic Classification of Antinuclear Antibody Patterns With Machine Learning

**DOI:** 10.7759/cureus.45008

**Published:** 2023-09-11

**Authors:** Baris Boral, Alper Togay

**Affiliations:** 1 Immunology, University of Health Sciences, Dr. Abdurrahman Yurtarslan Oncology Training and Research Hospital, Ankara, TUR; 2 Medical Microbiology and Immunology, Health Science University İzmir Tepecik Training and Research Hospital, İzmir, TUR

**Keywords:** neural network, hep-2, deep learning, indirect immunofluorescence, antinuclear antibody

## Abstract

Antinuclear antibodies (ANA) are important diagnostic markers in many autoimmune rheumatological diseases. The indirect immunofluorescence assay applied on human epithelial cells generates images that are used in the detection of ANA. The classification of these images for different ANA patterns requires human experts. It is time-consuming and subjective as different experts may label the same image differently. Therefore, there is an interest in machine learning-based automatic classification of ANA patterns. In our study, to build an application for the automatic classification of ANA patterns, we construct a dataset and learn a deep neural network with a transfer learning approach. We show that even in the existence of a limited number of labeled data, high accuracies can be achieved on the unseen test samples. Our study shows that deep learning-based software can be built for this task to save expert time.

## Introduction

Antinuclear antibodies (ANA) contain a wide range of autoantibodies targeting different antigens found in the cell nucleus. ANA is used as an important diagnostic marker in various autoimmune rheumatological diseases such as systemic lupus erythematosus, scleroderma, and polymyositis/dermatomyositis [1-3]. The indirect immunofluorescence (IIF) assay using human epithelial type 2 (HEp-2) cells is the most sensitive and gold-standard method used in the detection of ANA [4]. HEp-2 cells contain a wide variety of antigens in different cell cycles, allowing the detection of many clinically relevant autoantibodies [5]. For this reason, many morphological patterns emerge in IIF tests with HEp-2 cells. In order to reach a consensus on the diversity of ANA IIF morphological patterns, workshops are held regularly and consensus reports have been published under the name of the International Consensus on Antinuclear Antibody Patterns (ICAP), the first of which was in 2014-15 [6]. In this consensus report, the patterns are grouped under three headings: nuclear, cytoplasmic, and mitotic. Patterns under these three headings are numbered 1-28 with anti-cell (AC) code [6]. At the last meeting, 30 different HEp-2 IIF patterns were defined under four main headings: negative, nuclear, cytoplasmic, and mitotic [7].

ANA IIF testing requires expert knowledge for the classification of different patterns. They are subjective as different experts may label the same data differently. This subjectivity also comes from the low degree of standardization [8,9]. Even though there are dedicated trainings to achieve standardization, it remains an open problem. Furthermore, they are time-consuming for the expert as it increases the workload of the laboratory. Because of these reasons, there has been an increasing interest in the automatic classification of these patterns with computer programs. One of these efforts is EUROPattern [10] which can detect patterns with high precision. However, this software and other similar ones are expensive and may not be affordable, especially for small hospitals.

Recently, there has been significant progress in computer vision-related tasks thanks to the advances in machine learning especially in deep learning methods [11,12]. Through our study, we were interested in building an automatic tool that is affordable and accurate for ANA IIF testing. For this purpose, we built a dataset with microscope images of five different ANA patterns to learn a neural network machine-learning model for automatic analysis.

## Materials and methods

Indirect immunofluorescence assay

ANA was detected by the IIF method using commercially available HEp-20-10 cell substrates (Euroimmun, Lübeck, Germany). Testing and scoring were performed according to the manufacturer's instructions with an initial screening dilution of 1:100. After slide preparation, the test was scored according to the international consensus report on ANA specimens [5]. Extractable nuclear antigens (Euroline ANA Profile et Mi-2, Ku, DFS70, Euroimmun Lübeck, Germany) detected by the immunoblotting (IB) method were used according to the manufacturer's instructions. This method was used to determine Mi-2, Ku, DFS70, nRNP/Sm, Sm, SS-A, Ro-52, SS-B, Scl-70, Jo-1, CENP B, dsDNA, nucleosomes, histones, AMA-M2, and ribosomal P antibodies.

Dataset collection

**Figure 1:**
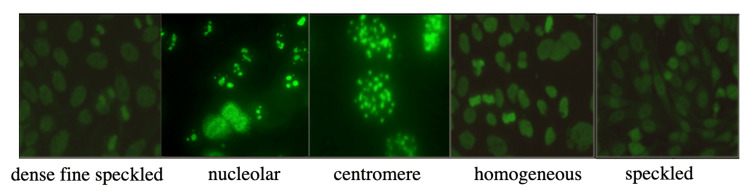
Examples from the training dataset.

With the procedure described above images were collected that belong to five different classes: centromere, nucleolar, homogeneous, speckled, and dense fine speckled (anti-DFS70) which are some of the most common types. Images were labeled with these classes by two immunology experts with more than 10 years of experience. In addition, the IIF patterns used in the study were confirmed by the IB method. Examples of these image-ground truth pairs are given in Figure 1. Our dataset contains 200 images, and we used 130 of them for training and 70 for testing.

Machine learning model

**Figure 2:**
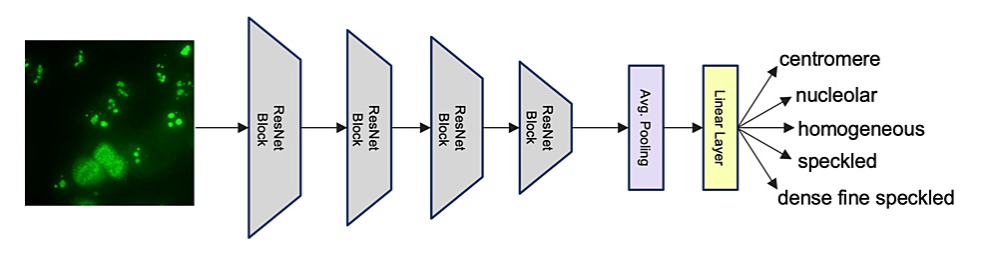
The architecture of the neural network model used in the classification task.

We ran experiments with convolutional neural networks given their success in computer vision tasks. One problem with these networks is that they are data-hungry and require a lot of labeled data for their training. However, it has been shown that one can use pre-trained models and fine-tune these models for different tasks when less data is available which is called transfer learning. We used the transfer learning approach by utilizing models that were trained on the ImageNet dataset [13]. Specifically, we used the ResNet18 [12] model which is a popular architecture for image classification tasks. It employs four ResNet blocks as shown in Figure 2.

Each block consists of two convolutional layers followed by a rectified linear unit activation function. The input coming to a block is skipped to the output by summation. These skip connections are referred to as residual layers and give their name to the ResNet architectures. After these blocks extract features from image inputs, there is an average pooling layer that decreases the spatial resolution of features to 1x1. We added a linear layer that projected the averaged features to a dimension that fitted our classification task. Specifically, the linear layer outputs five neurons corresponding to the number of possible outcomes of our classification problem. We used the cross-entropy loss function as our objective function and optimized the learnable parameters of the neural network with stochastic gradient descent.

As mentioned above, the final layer was customized to the classification task. We initialized the parameters of the final layer with random variables and the rest of the network was copied from the pre-trained model. Then, we trained the network on our dataset by using the PyTorch machine learning framework. Images were resized to 256 dimensions and random crops of 224x224 squares were taken during training to increase the effective number of images. We trained models for 25 epochs with a learning rate of 0.001 and momentum of 0.9.

## Results

The performance of the model was validated on 70 images. It achieved an accuracy of 77.14%. The confusion matrix for the validation set is shown in Table 1.

**Table 1:** Confusion matrix of our model on the test set

	Prediction					
Ground-truth		Dense fine speckled	Speckled	Homogeneous	Nucleolar	Centromere
	Dense fine speckled	19	2	1	0	0
	Speckled	5	6	0	0	1
	Homogeneous	5	0	15	0	0
	Nucleolar	0	1	0	5	0
	Centromere	0	0	0	1	9

The diagonal shows the correct predictions referred to as true positives. Rows represent the ground-truths and columns represent the predictions. Among 21 dense fine-speckled samples, our model correctly predicts 19 of them, and 2 of them are predicted as speckled and 1 of them as homogeneous. The most confused class is speckled. Among 12 samples, half of them are correctly predicted whereas 5 of them are wrongly predicted as dense fine speckled and 1 as centromere. Among the 20 homogeneous ground-truth samples, 15 of them are correctly classified and 5 of them are misclassified as dense fine speckled. Among 6 samples of nucleolar, only 1 of them is misclassified as speckled, and among 10 samples of centromere, only 1 sample is misclassified as nucleolar.

**Figure 3:**
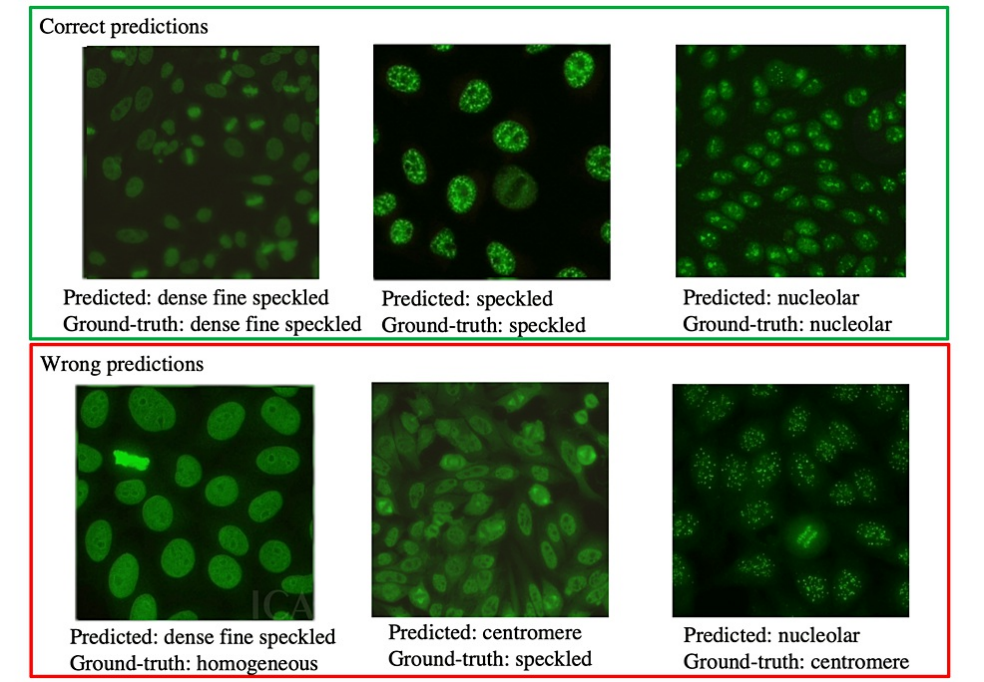
Example images from the test set and the predictions of our model.

In our study, we see that our method correctly classifies the majority of the samples. Some of those correct as well as wrong predictions are shown in Figure 3.

## Discussion

Machine learning-based automatic tools are becoming useful in many tasks. They have the advantage of being very fast and scalable across multiple computers. On the other hand, their reliability is a very important topic. In this work, our method even though trained on a small-scale dataset achieved 77.14% accuracy which shows deep learning-based artificial intelligence models are promising tools for this task. Currently, there are automated ANA IIF testing systems available [3]. These systems have the ability to reduce labor time, however, they are not as accurate as the manual evaluation yet. Therefore, studies on this topic are essential to achieve the most accurate results. 

We observed that the artificial intelligence model makes mistakes while detecting speckled, homogeneous, and dense fine-speckled (DFS-70) patterns. This is a predictable error because the DFS-70 pattern resembles a homogeneous pattern with positive metaphases and a speckled pattern with involvement in the interphase cell phase. Therefore, it can misidentify homogeneous and speckled patterns as dense fine speckled. Our algorithm had the most difficulty distinguishing between DFS-70 and speckled pattern, misestimating 5 patients in the DFS-70 pattern. This is because the DFS-70 pattern is included in the dense fine speckled pattern, which is a subgroup of the speckled pattern. These two patterns are quite similar to each other, and their distinction can be made from the involvement in metaphases. To avoid such misidentifications, artificial intelligence should conclude by evaluating the interphase and metaphase phases together. 

Previously, computer vision algorithms have been used to analyze ANA IIF images. For example, Otsu thresholding is used for segmentation of IIF images [14,11]. However, recently in all image-related tasks, deep learning-based methods replaced traditional computer vision algorithms due to their efficiency and high accuracy. Concurrent with our work, there have been other studies that use deep learning on HEp-2 specimen medical images. Anaam et al. show that mitotic cells can be detected in those images with high precision [15]. Their method requires bounding box annotations on the images which is expensive to obtain. Xie et al. present methods for the segmentation and classification of ANA IIF images [11]. In their work, for the segmentation and feature extraction, traditional computer vision techniques are used but for classification similar to our work a deep learning algorithm is proposed. Our work is different from theirs because we propose an end-to-end deep learning-based solution for ANA pattern classification. In our system, both feature extraction and classification are achieved by a single neural network. We also show that good results can be achieved with small-size datasets. Being able to learn classification models with a small number of labeled datasets is important to scale to different laboratory and imaging setups. 

## Conclusions

This paper presents a study for the automatic classification of ANA IIF patterns. Classification of those patterns is important as they are the diagnostic markers in various autoimmune rheumatological diseases. Currently, these classifications require human experts and they put a significant amount of workload on the experts which makes automatic classifiers on this task to be highly desirable. In our study, we construct a dataset and learn a deep neural network with a transfer learning approach and show that even in the existence of a limited number of labeled data, high accuracies can be achieved on unseen test samples. Our study shows that deep learning-based software can be built for this task to save expert time.
